# Human kinematic, kinetic and EMG data during different walking and stair ascending and descending tasks

**DOI:** 10.1038/s41597-019-0323-z

**Published:** 2019-12-06

**Authors:** Tiziana Lencioni, Ilaria Carpinella, Marco Rabuffetti, Alberto Marzegan, Maurizio Ferrarin

**Affiliations:** IRCCS Fondazione Don Carlo Gnocchi, Milano, Italy

**Keywords:** Biomedical engineering, Motor control, Electromyography - EMG

## Abstract

This paper reports the kinematic, kinetic and electromyographic (EMG) dataset of human locomotion during level walking at different velocities, toe- and heel-walking, stairs ascending and descending. A sample of 50 healthy subjects, with an age between 6 and 72 years, is included. For each task, both raw data and computed variables are reported including: the 3D coordinates of external markers, the joint angles of lower limb in the sagittal, transversal and horizontal anatomical planes, the ground reaction forces and torques, the center of pressure, the lower limb joint mechanical moments and power, the displacement of the whole body center of mass, and the surface EMG signals of the main lower limb muscles. The data reported in the present study, acquired from subjects with different ages, represents a valuable dataset useful for future studies on locomotor function in humans, particularly as normative reference to analyze pathological gait, to test the performance of simulation models of bipedal locomotion, and to develop control algorithms for bipedal robots or active lower limb exoskeletons for rehabilitation.

## Abstract (summary)

**Table:** 

Measurement(s)	kinematic data • kinetic data • electromyography • locomotor behavior trait • motion analysis data
Technology Type(s)	stereophotogrammetry • dynamometric force platform • electromyography device • multifactorial motion analysis
Factor Type(s)	type of locomotor task • age • sex • gait speed • body height • body weight
Sample Characteristic - Organism	Homo sapiens

Machine-accessible metadata file describing the reported data: 10.6084/m9.figshare.11112980

## Background & Summary

Locomotion, the movement of the body from one place to another, is a fundamental task for animal life and it is performed through complex interactions among the neuro-muscular, skeletal and sensory systems^1^. The way locomotion is realized highly depends on the environment where it is performed. Humans, like all terrestrial mammals, use legged locomotion requiring the continuous and alternate repositioning of each limb on the ground. Although legged locomotion is a dynamically unstable process, thus not easy to be learned and maintained at all stages of life, it offers some advantages like the possibility to be performed on different kinds of terrain, to overcome obstacles, to climb/descend stairs and to make sharp turns^2^. The main conditions of human locomotion are walking and running, the first being the most common one adopted during daily-life activities, with an average daily step count of about 9500 in adults^3^.

When problems arise due to abnormal functioning of one or more components of the motor system, human movements are altered and walking becomes less efficient, difficult or even impossible to be performed^4^. Also physiological changes associated to aging may worsen deambulation^5^. Rehabilitation approaches and assistive walking devices, e.g. canes, crutches, orthoses, can (at least partially) solve such problems and/or support patients to improve locomotion.

An important aspect in this context is the objective quantitative assessment of walking abnormalities in order to develop new rehabilitation protocols and assistive devices, to tailor/personalize them to each subject, and to verify their efficacy over time^6^. A key point in this process is the availability of normative data measured on healthy persons aimed at quantifying possible deviations of the walking pattern of a given subject from the physiological profiles^7,8^.

Three types of data are traditionally considered to fully describe human walking: kinematic, kinetic and electromyographic (EMG)^7^. Kinematic data include displacement and orientation of body segments, joint angles and spatio-temporal gait parameters. Kinetic data include ground reaction forces (GRF), lower limb joint mechanical moments and powers, kinetic and potential energy. Muscle activation patterns are analyzed through the electrical signals (EMG) associated to muscular fibre contraction, that can be recorded noninvasively through surface electrodes attached on the skin over muscle bellies.

The present dataset has been collected using the state-of-the-art instruments of a gait analysis laboratory, which include: a TVC-based stereophotogrammetric system, a set of passive reflective markers, dynamometric force platforms and a wireless multichannel EMG recording system.

Few databases including kinematics, kinetics and EMG data of healthy subjects during locomotor tasks and upper limb exercises have been published^7–14^. Most databases reporting lower limb data, usually provide between-subject average profiles in numerical format^7–9^, while single subject data are made available in one database only which provides kinematic and force plate data but not EMG signals^12,13^. Moreover, most of published databases concern only level walking, and did not consider other locomotor tasks, such as toe- and heel-walking^15^ and stair negotiation^16^, that can be more sensitive to mild alterations typical of early-stage neuromuscular diseases. In particular, compared to plain walking, toe- and heel-walking challenge balance and imply a stronger distal activity of ankle muscles, while stair negotiation implies larger amounts of energy to be produced during ascending or dissipated during descending, and asks for larger range of motion and moment of hip and knee joints^17^. Moreover, it is well known that walking speed has apparent effects on kinematics, kinetics and muscle recruitment^8^ and that increased speeds are able to disclose locomotor anomalies^18^.

Based on the above considerations, in the present paper we report a comprehensive dataset of kinematic, kinetic and EMG individual data measured on 50 healthy subjects of different ages (young, adult and elderly) during the following conditions: level walking at different velocities, toe- and heel-walking, and stairs ascending and descending. Possible applications of the data here reported are: providing age- and speed-matched normative reference for the analysis of altered gait in individual patients, developing/optimizing lower limb prostheses^19^, developing/testing simulation models of bipedal locomotion^20^, and developing control algorithms for bipedal robots^21^, active exoskeletons^22,23^ or model-based Functional Electrical Stimulation systems^24^ for motor rehabilitation.

## Methods

### Participants

Fifty healthy subjects (25 males, 25 females, age range: 6–72 years, body mass: 18.2–110 kg, body height: 116.6–187.5 cm) were included in the study. All of them reported no known locomotor disorders or other health issues which could affect their motor performance. Age, gender, body weight and body height of each participant are reported in the data set.

Before engaging in experiments, each subject was comprehensively briefed about the procedure, introduced to the experiment and informed of any potential risks. We required participants to sign an informed consent form. The study and experiments were carried out in accordance with principles of the Declaration of Helsinki and approved by the Institutional Research Ethics Committee of Fondazione Don Carlo Gnocchi.

### Instrumentation and subjects preparation

Data were acquired in the LAM (Laboratory for Movement Analysis) of the Biomedical Technology Department of Don Carlo Gnocchi Foundation Scientific Institute of Milano, Italy. The laboratory is equipped with a 9-camera motion capture system (SMART system, BTS, Garbagnate Milanese, Italy), two force platforms (Kistler, Winterthur, Switzerland) and a 8-channels wireless EMG recording system (ZeroWirePlus, Cometa, Bareggio, Italy).

Synchronous data acquisition was managed by the proprietary software of the motion capture system (SMART Capture, version 1.10, BTS, Italy). The markers trajectories were recorded at either 60 Hz or 200 Hz, force plate data at 800 Hz or 960 Hz, EMG data at 800 Hz, 960 Hz or 1000 Hz. Sampling frequencies are indicated in the data files.

The calibrated volume of the SMART-DX system was about (5 × 3 × 2) m^3^, within which the 3D coordinates of the retro-reflective markers could be reconstructed with an accuracy of less than 1 mm in all directions. The calibration of cameras’ and platforms’ positions was performed before each acquisition session, following the standard procedure described by the producer of the motion capture system.

**Fig. 1:**
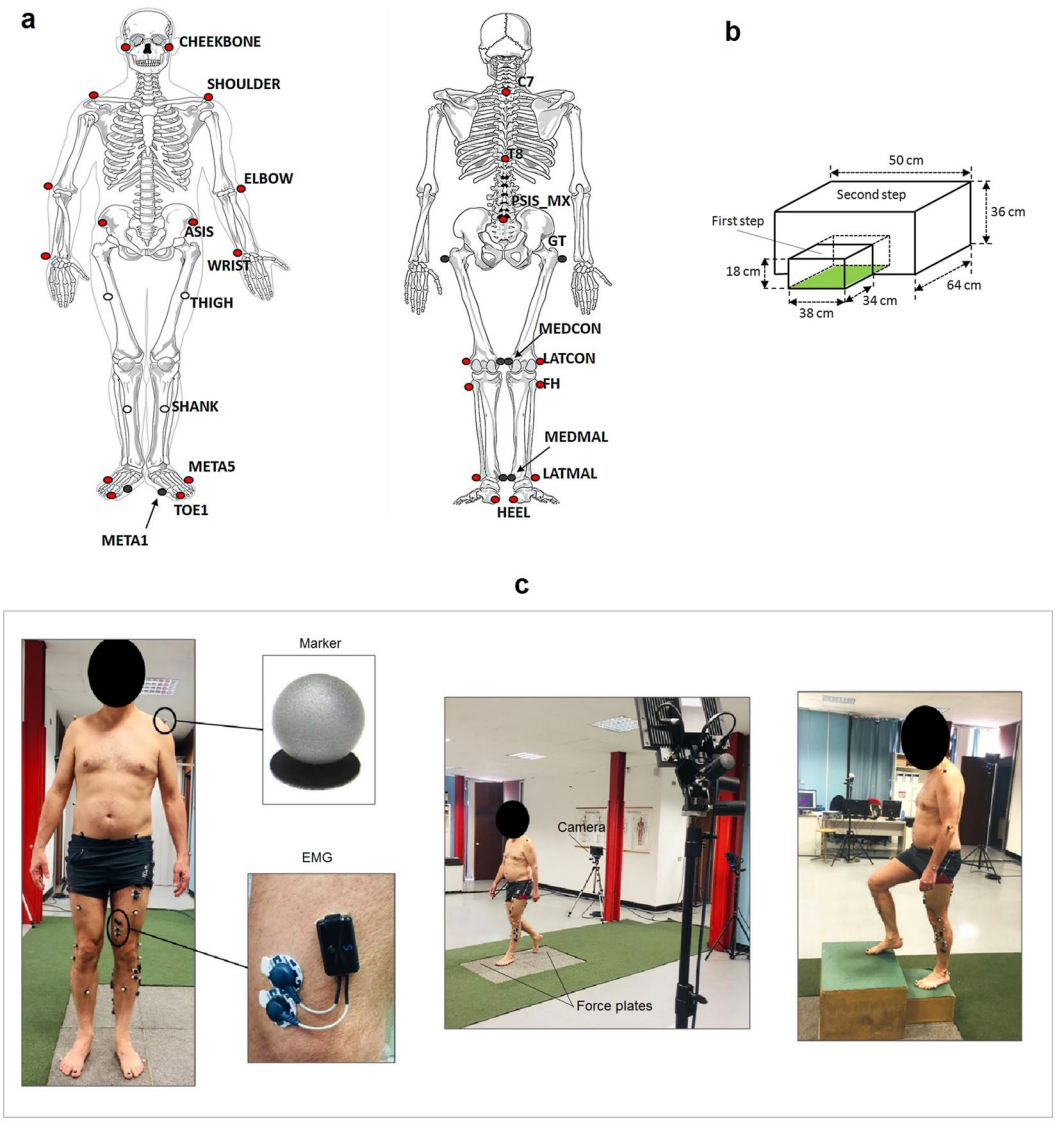
Experimental set-up. (**a**) Marker set of LAMB protocol, which includes a total of 29 markers: 25 anatomical markers (red dots) and 4 technical markers (white dots). Skeleton figures are taken from another source (https://pixabay.com/en/skeleton-human-skeletal-anatomy-41548/ and https://pixabay.com/en/skeleton-human-skeletal-anatomy-41550/). A preliminary calibration phase requires a static trial with additional 8 anatomical markers (grey dots), which are removed during the dynamic trials. The label of all markers are indicated. ASIS: anterior superior iliac spine; META1 and META5: first and fifth metatarsal heads; PSIS_MX: midpoint between right and left posterior superior iliac spines; GT: great trochanter; MEDCON and LATCON: medial and lateral femoral condyles; FH: fibular head; MEDMAL and LATMAL: medial and lateral malleoli. **(b)** Schematic drawing of the staircase used for step ascending/descending tasks (dynamometric platform is colored in green). **(c)** Pictures of a subject during the experiment equipped with markers and electrodes for EMG recording (see blowups). From left to right: standing, walking and step ascending trial.

At the beginning of each experiment, the subject was equipped with the LAMB total-body marker set^25^, which includes 29 retro-reflective markers (12 mm diameter) on head, upper limbs, trunk, pelvis and lower limbs (red and white dots in Fig. 1a). As required by the LAMB protocol, 8 additional markers were placed on great trochanters and medial part of the lower limbs (gray dots in Fig. 1a). These markers were used during the preliminary static calibration trial and were removed during the dynamic trials.

Subjects were required to wear tight clothes or swimsuit and markers were attached on the skin above bony landmarks with double-sided adhesive tape. Surface EMG signals were recorded using pre-amplified self-adhesive Ag-AgCl electrodes (Medtronic Kendall, diameter: 24 mm, diameter of the active part: 10 mm, bipolar configuration, interelectrode distance: 20 mm) and were band-pass filtered (10 Hz–400 Hz) before sampling to reduce the aliasing effect.

EMG signals were recorded unilaterally, on the dominant side, from the following muscles tibialis anterior (TA), soleus (SO), gastrocnemius medialis (GM), peroneus longus (PL), rectus femoris (RF), vastus medialis (VM), biceps femoris (BF) and gluteus maximus (GMax). Electrodes were located on the skin according to SENIAM recommendations^26^. The recording sites were shaved to remove any hair and subsequently cleaned with an alcohol solution and allowed to dry. The signals were checked for quality and sensors locations were adjusted as necessary.

### Experimental procedure

For each subject, the acquisition session included a static calibration phase and, subsequently, a dynamic one including five locomotor tasks: walking at different speeds, toe-walking (T), heel-walking (H), step ascending (U) and step descending (D). All tasks were performed barefoot.

The static calibration phase included two static trials: the calibration and the standing trial. The calibration trial, performed in an erect posture such that all markers are visible, is used to derive anthropometric measurements (e.g. femoral length) and to calibrate the position of the great trochanters and the medial part of the body, since the latter are hardly visible during locomotor movements. The standing trial, performed in the natural erect posture of each subject, is recorded to compute reference values for kinematic data during locomotion.

After the calibration phase, the 8 anatomical markers placed on great trochanters and medial lower limbs were removed and the dynamic locomotor tasks were acquired.

For the walking task, we initially asked the subjects to walk five trials at their natural speed (N). Hence we asked to perform the following ten trials while progressively increasing (first 5 trials) or decreasing (latter 5 trials) their speed. We gave no precise indications about gait speed or cadence in order not to induce gait alterations.

During step ascending (U) and descending tasks (D), a custom-made staircase consisting of two wooden steps was used. As shown in Fig. 1b, the first step was positioned on one force platform to provide GRF data, after removing the step weight through the platform reset procedure preliminarily performed. The second step was a structure with a bridge shape that did not interfere mechanically with the first step and leaned on the ground outside the force platform. During U, subjects were required to start from ground level and stop with both feet on the second step. Correspondingly, during step descending (D), the subjects started standing on the second step, and stopped on the ground beyond the first step. The subjects performed toe-walking (T), heel-walking (H) and step negotiation (U and D) at their self-selected speed. We asked the subjects to repeat each task five times. A total of 35 locomotor trials were recorded for each participant, during a single acquisition session lasting about 1 hour. Only data from trials correctly completed (i.e. correct foot-strike on force platforms and absence of evident artifacts) and without technical problems in data collection are reported.

Figure 1c shows a subject equipped with markers and EMG electrodes during the recording session.

### Data elaboration

**Fig. 2:**
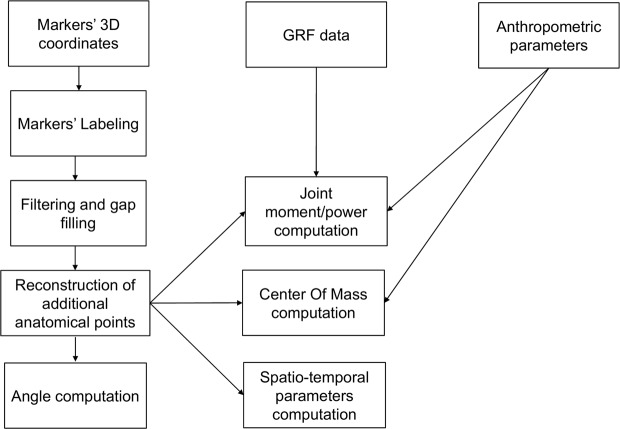
Data processing. Flowchart of data elaboration.

The flowchart of data elaboration is shown in Fig. 2.

After data acquisition, the 3D markers’ coordinates were preprocessed using SMART Tracker software (ver. 1.10) which allowed the labeling of each marker with a specific name, according to Fig. 1a. This operation defined the anatomical location of each marker. Moreover, the same software performed the computation of the 3D trajectories of each marker.

After the labeling and 3D reconstruction procedures, kinematic data were further elaborated using a custom software implemented with MATLAB R2017b (The Mathworks Inc., Natick, MA).

In particular, the 3D coordinates of labeled markers of the lower limbs and pelvis were low-pass filtered (5^th^ order, zero-lag, Butterworth filter) at a frequency equal to 6 Hz according to Rabuffetti *et al*.^25^, and the trajectory gaps were filled through spline interpolation in case of short periods (maximum 10% of the entire stride) of markers’ masking as described in technical validation section.

The 3D coordinates of all acquired markers are made available in the data record associated to this paper, as described in the “Data records” section, together with dynamometric platform data (3D ground reaction forces and torques, 3D coordinates of Center of Pressure (CoP)) and EMG signals. It must be noted that CoP coordinates reported in the data record are referred to the global reference frame of the optoelectronic system. Moreover, as regard step ascending and descending tasks, the reported CoP coordinates are referred to the plane of the platform, that was 18 cm (first step height) below the actual contact surface between foot and step.

To provide the interested reader with standard 3D kinematic and dynamic gait data, pelvis orientation angles, hip, knee and ankle joint angles, moments and powers, and 3D coordinates of the body center of mass have been computed according to the LAMB model and reported in the data record too.

In the following paragraphs a summary description of the LAMB model is reported, while a detailed characterization can be found in Rabuffetti *et al*.^25^.

The LAMB model is based on the notion that the availability of the 3D coordinates of three markers and/or estimated points on each segment allows for the identification of local reference frames.

Firstly, the anthropometric parameters of each subject were computed from markers’ positions recorded during the calibration trial. In particular, the leg length was estimated as the Euclidean distance between ASIS and LATMAL markers, while the pelvis depth as the component of the ASIS-GT distance onto the antero-posterior pelvis axis passing through the PSIS_MX and the midpoint between right and left ASIS markers.

Markers’ trajectories and anthropometric parameters were used to estimate the hip joint center (HJC) following Davis *et al*.^27^, and to reconstruct the additional anatomical markers in the dynamic trials, following the calibration procedure described by Cappozzo *et al*.^28^. In particular, GT and MEDCON were reconstructed using HJC, THIGH and LATCON markers, MEDMAL using FH, SHANK and LATMAL markers, and META1 using MEDMAL, LATMAL and META5 markers.

Secondly, the local reference frame of each segment was computed. The pelvis reference frame, identified according to Davis *et al*.^27^, was defined by a forward-oriented X-axis passing through the PSIS_MX and ASIS midpoint, an upward-oriented Y-axis perpendicular to the PSISs/ASISs plane, and a Z-axis computed as the cross-product of X and Y axes. The thigh reference frame was defined by an upwards longitudinal Y-axis passing through the HJC and the midpoint between LATCON and MEDCON (i.e. knee joint center, KJC), a forward-oriented X axis perpendicular to the plane identified by the thigh longitudinal Y-axis and by the LATCON-MEDCON vector, and a Z-axis that is the cross-product of X and Y. The shank reference frame included an upwards longitudinal Y-axis passing through the KJC and the ankle joint centre (AJC), the latter estimated as the midpoint between LATMAL and MEDMAL, a forward X-axis perpendicular to the plane identified by the shank longitudinal Y-axis and by the LATMAL-MEDMAL vector, and a Z-axis that is the cross-product of X and Y. Finally, the foot reference frame was characterized by a longitudinal X-axis passing through the AJC and the midpoint between META1 and META5, an upwards Y-axis perpendicular to the plane defined by the foot longitudinal X-axis and the META5-META1 vector, and a Z-axis computed as the cross-product between X and Y.

Angular variables were computed, according to Grood & Suntay^29^ and Wu *et al*.^30^, by considering the local reference frames of two adjacent anatomical segments in the case of joint angles, and by considering the absolute functional frame and the segment local frame in the case of pelvis orientation angles.

Joint moments were computed following a free body approach and using published estimates of segments inertial characteristics based on subjects’ anthropometry^31^. The resulting moment vector was presented by the components relative to the local frame of the proximal segment. Joint powers were computed, for each joint, multiplying the flexion/extension joint moment with the first derivative of the flexion/extension joint angle. Joint moments and powers were normalized to the body mass of each subject, reported in the data record.

The 3D coordinates of the center of mass of the whole body were computed following a segmental analysis method^32^. In particular, the positions of the centers of mass of each body segment were computed from the 3D coordinated of markers and/or reconstructed points and from subjects anthropometric features. The mass of each segment was estimated following Zatsiorsky *et al*.^31^. Hence, the location of the whole body center of mass was computed frame by frame by using a weighted average of segmental center of mass based upon the segment mass fractions^7,33^.

For each trial, foot-strikes were defined from platform data as the first samples with presence of GRF data. Two consecutive foot-strike instants correspond to the initiation (t_start_) and termination (t_end_) of the stride. Only strides including one step on a force plate were considered.

The data reported in the present study includes, for each trial, the portions of kinematic, dynamic and EMG signals between t_start_ and t_end._ In the case of angles, joint moments and powers, the signals were time normalized to 100 points as a percentage of stride duration (t_end_–t_start_).

Gait speed was computed as the ratio between the linear distance traveled by the HJCs’ midpoint during a stride and the stride duration. Stride length was computed as the linear distance traveled by AJC during a stride. The cadence (i.e. step/min) was calculated as 60/(0.5*stride duration). In data records also the step width is provided as the lateral distance between the AJC of the supporting foot and the contralateral one.

## Data Records

All published data are de-identified and data files are available from *FigShare*^34^ under the terms of Attribution 4.0 International Creative Commons License (http://creativecommons.org/licenses/by/4.0/).

The data set consists of multiple files, each of which corresponds to the data of one subject Each file is organized with the following naming convention: SubjectX.mat, where X is the subject number (from 1 to 50), and .mat is the format file (a MAT-file is the data file format of MATLAB® software).

### SubjectX.mat

The SubjectX.mat file holds a structure (*s*) which contains the subject’s anthropometric variables (Age [years], Gender [M/F], Body Height: BH [cm], Body Mass: BM [kg]), the sampling rate [Hz] of the kinematic, kinetic, and EMG data (KinFreq, GrfFreq, EMGfreq), the dominant leg from which the EMG signals were acquired (EMGSide [RX/LX]), the names of the angles (AngVarName), moments (MomVarName), powers (PwrVarName), GRF variables (GrfVarName), EMG signals (EmgVarName), and markers (MarkerVarName). The structure *s* contains also two fields (Data and StandingData) which report, respectively, the data of the locomotor trials and the standing trial. In details, these fields are described in Table 1. The length L of *s.Data* [L = length(s.Data) in MATLAB® language] represents the number of reported trials.

**Table 1:** Contents of *s.Data* and *s.StandingData* structures in SubjectX.mat files.

Field of *s.Data* structure	Description
.Foot	Indicates the trailing foot (i.e. the foot on the force plate), RX (right) or LX (left)
.TimeStampKin	The timestamp of the foot-strike in the kinematic time vector, indicating the beginning of the stride (unit: s)
.TimeStampGrf	Same as *.TimeStampKin* in the kinetic time vector related to the ground reaction force (unit: s)
.TimeStampEMG	Same as *.TimeStampKin* in the EMG time vector (unit: s)
.Task	Indicates the type of trial (Walking, HeelWalking, ToeWalking, StepDown, StepUp)
.Marker	Matrix (3·Nmarker × N). It contains the 3D trajectories of the markers. The rows are the X, Y, Z coordinates of the markers named in the variable *s.MarkerVarName*. The columns are the N sample points (unit: m)
.EMG	Matrix (8 × N). It contains the EMG data. The rows are the channels named in the variable *s.EMGVarName*. The columns are the N sample points (unit: mV)
.Grf	Matrix (9 × N). It contains the Grf data. The rows are the signals named in the variable *s.GrfVarName*. The first three rows are relative to the ground reaction force vector (unit: N), the second three rows are relative to the position of the application point of the ground reaction force (center of pressure, unit: mm), while the last three are relative to the ground reaction torque vector (unit: N*m). The columns are the N sample points.
.speed	Averaged speed (unit: m/s)
.strideLength	Length of the stride (unit: m)
.stepWidth	Width of the stride (unit: m)
.cadence	Cadence (unit: step/min)
.Ang	Matrix (12 × 101). The rows are the pelvis orientation and joint angles corresponding to the anatomical planes reported in the variable *s.AngVarName*. The columns are the sample points expressed as percentage of the stride duration (unit: deg)
.Mom	Matrix (9 × 101). The rows are the joint moments corresponding to the anatomical planes reported in the variable *s.MomVarName*. The columns are the sample points expressed as percentage of the stride duration (unit: Nm/kg)
.Pwr	Matrix (3 × 101). The rows are the joint powers corresponding to the sagittal plane reported in the variable *s.PwrVarName*. The columns are the sample points expressed as percentage of the stride duration (unit: W/kg)
.Com	Matrix (3 × 101). The rows are the X, Y, Z coordinates of the trajectory of the Center of Mass. The columns are the sample points expressed as percentage of the stride duration (unit: m)
**Field of** ***s.StandingData*** **structure**	**Description**
.Marker	Matrix (3·Nmarker × 1). It contains the averaged values of the 3D locations of the markers during the standing trial. The rows are the X, Y, Z coordinates of the markers named in the variable *s.MarkerVarName*. The columns are the N sample points (unit: m)
.Ang	Matrix (12 × 1). It contains the averaged values of the joint angles during the standing trial. The corresponding anatomical planes are reported in the *s.AngVarName* (unit: deg)

## Technical Validation

### Kinematic and EMG data

The calibration of cameras’ and platforms’ positions was performed before each acquisition session, following the standard procedure described by the manufacturer of the motion capture system. Moreover, before each trial, the force plates were reset to remove their offset through the system’s acquisition software.

Markers and EMG electrodes were positioned by experts gait analysts (Authors TL and AM) according to published protocols^25,26^. The EMG signals were checked for quality and sensor locations were adjusted if necessary. In cases where the signal was persistently noisy, even after skin preparation, an abrasive skin gel was applied to remove dead cells and reduce impedance.

Marker labelling and 3D trajectory reconstruction were performed using the proprietary software of the motion capture system (SMART Tracker, version 1.10) and the results were visually checked trial by trial. Among the 1615 strides that constitute the dataset, only 0.6% of the recorded frames were missing data. For each stride, gap filling was applied to the markers of the pelvis and trailing limb (i.e. the supporting leg), only if a marker was missing for a maximum period equal to 10% of the entire stride. This threshold was chosen after a preliminary analysis involving (i) the artificial cutting of signals portions of different lengths (1%, 2%, 5%, 10%, 25%, 50% of stride duration), (ii) the portions’ filling through spline interpolation (interp1 with SPLINE option in MATLAB® language), and (iii) the calculation of the root mean square error (RMSE) between the interpolated signals and the original ones. This procedure was applied to four markers (Asis_Rx, Psis_Mx, LatMal_Rx, Meta5_Rx) at four time frames (foot-strike and toe-off, both right and left). The median RMSE value was lower than 0.40 mm (inter-quartile range: 0.26 mm–0.89 mm) until the number of missing data was less than 10% of the stride duration. By contrast, at a percentage of 25%, the RMSE median value was 2.0 mm (interquartile range: 1.04–4.44 mm). Based on this analysis, it was concluded that the adopted interpolation algorithm efficaciously works for holes up to 10% of stride duration.

A picture of the missing data remaining after the gap filling procedure is provided in SupplementaryFile01.xlsx and SupplementaryFile02.xlsx (see Supplementary Information). In particular the SupplementaryFile01.xlsx summarizes the amount of missing values present in each matrix contained in the structure *s.Data(i)* for each trial *i* (i.e. .Marker, .Com, .Ang, .Mom, .Pwr, .EMG, .Grf). In addition, the SupplementaryFile02.xlsx shows the amount of missing values for each marker.

### Reliability of LAMB protocol

The LAMB protocol has been extensively used to analyze different locomotor tasks in healthy subjects^9,23,35^ and persons with motor disturbances^6,15,17,36–39^.

The marking reliability of LAMB has been demonstrated comparable to other protocols^25^. In particular, the between-operator marking variability was between 4.2 mm and 20.8 mm, in line with data from Rabuffetti *et al*.^40^ (range: 6.7 mm–28.7 mm) and Della Croce *et al*.^41^ (range: 13.4 mm–20.5 mm). Also the within-operator marking variability (range: 3.7 mm–18.9 mm) was comparable to previously published data (range: 6.5 mm-17.9 mm)^41^. The between-operator mean (standard deviation) absolute variability of kinematic and kinetic outcomes was 7.3 (3.7) deg for angular temporal profiles, 0.08 (0.06) Nm/kg for moments, and 0.30 (0.15) W/kg for power data^25^. These values are comparable with those reported by Ferrari *et al*.^42^ [15.6 (9.1) deg for angles, 0.18 (0.08) Nm/kg for moments] and by Leardini *et al*.^43^ [8.6 (4.2) deg for angles, 0.14 (0.09) Nm/kg for moments]. The average minimum correlation coefficient for gait data was 0.907 for angles, 0.857 for moments and 0.882 for powers suggesting a high similarity in the shape of different temporal profiles^25^. These values are in line with those previously reported^42^. Importantly, the variability indexes were comparable among tasks^25^, suggesting that the LAMB protocol is reliable and suitable for the analysis of different locomotor acts.

In addition, the same locomotor tasks (N, T, H, U, D) reported in the present data set and acquired through the LAMB protocol have shown a good inter-session reliability in a population of subjects with neuromuscular disease^15^.

**Fig. 3:**
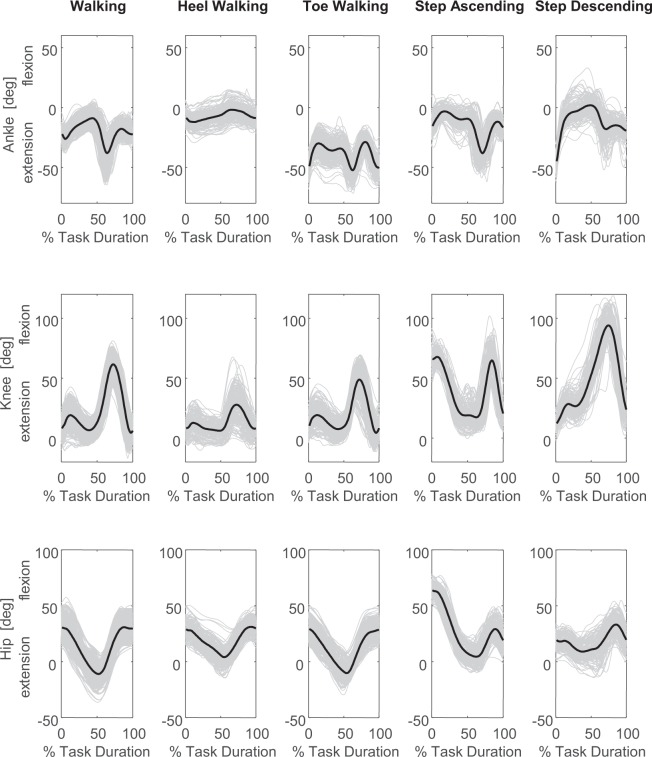
Lower limb joint angles of all fifty subjects and all trials during level walking, toe-walking, heel-walking, step ascending and step descending. Each grey line represents the individual joint angles in the sagittal plane during a cycle and the black line represents the group average curve.

**Fig. 4:**
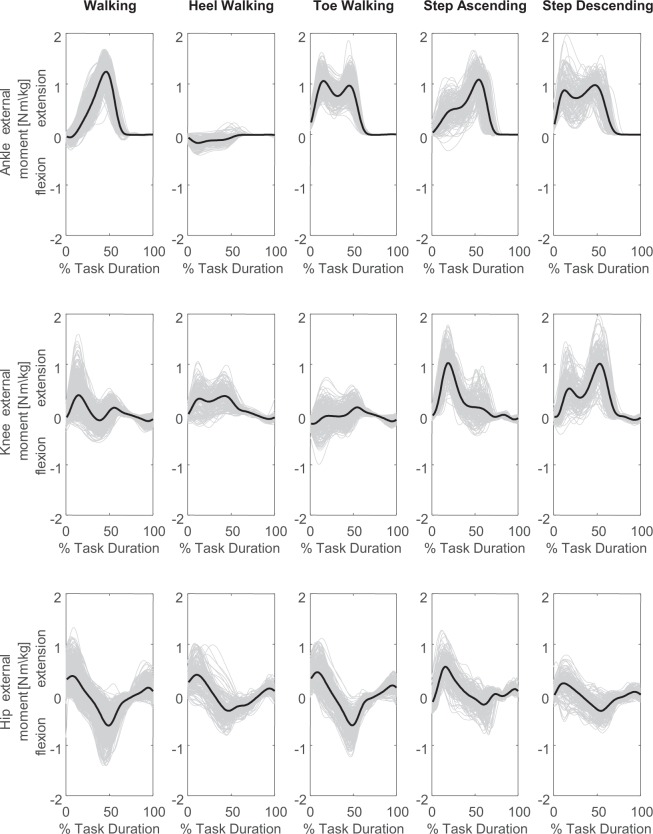
Lower limb joint moments of all fifty subjects and all trials during level walking, toe-walking, heel-walking, step ascending and step descending. Each grey line represents the individual joint moments in the sagittal plane during a cycle and the black line represents the group average curve.

The hip, knee, and ankle joint angles and moments in the sagittal plane are shown in Figs. 3 and 4 respectively, for all 50 subjects during all reported trials.

### Comparison with published reference dataset

Present study population includes a sub-sample of 21 young subjects (mean ± SD age: 10.9 ± 3.2 years, range 6–17 years) that closely matched the sample analyzed by Schwartz *et al*.^8^ (mean ± SD age: 10.5 ± 3.5 years). In order to compare the two datasets, we selected the walking trials (performed by the 21 young subjects) that were characterized by a normalized gait speed (Hof method^44^) between 0.36 and 0.50, according to the criteria adopted by Schwartz *et al*.^8^ Hence, we computed the Pearson’s correlation coefficient (r) between the average curves of the two data sets, as proposed by Ferrari *et al*.^43^. The results showed a high correlation between our data, recorded with LAMB protocol, and the ones of Schwartz *et al*.^8^, acquired with Vicon Plug-in Gait protocol. In particular, r values ranged from 0.74 to 0.99 for kinematic variables, from 0.69 to 0.99 for kinetics, and from 0.83 to 0.92 for EMG profiles.

## Usage Notes

A script (walking_fig.m) is provided to assist with the reuse of the data. The script is deposited together with data files in *FigShare*^34^. The walking_fig.m file must be placed in the same directory containing the data files and must be run using MATLAB Software. The script contains an example on how to read and visualize angles, moments and powers of ankle, knee and hip joints of a single subject (Subject 6) executing straight line walking at different speed.

## Supplementary information

Supplementary Table 1

Supplementary Table 2.

## Data Availability

In addition to the methods here presented, the instructions for reproducing the LAMB protocol are fully described in Rabuffetti *et al*.^25^. Moreover, the code used for computing the angular variables is provided together with data files in *FigShare*^34^. In details, the MATLAB function “CalcAngle.m” computes the angles in the three anatomical planes, starting from the reference frames of two adjacent body segments.
